# Patterns and Predictors of *Candida auris* Candidemia with Multidrug-Resistant Bacterial Co-Infections: Results from the CANDI-MDR Study

**DOI:** 10.3390/jof11060407

**Published:** 2025-05-25

**Authors:** Karolina Akinosoglou, Katerina Skintzi, Ioannis Chandroulis, Eleni Polyzou, Argiro Siapika, Foteini Fligkou, Fotini Paliogianni, Charalambos Gogos, George Dimopoulos

**Affiliations:** 1 Department of Medicine, University of Patras, Rio, 265 04 Patras, Greece; katrinski80@gmail.com (K.S.); polyzou.el@gmail.com (E.P.); fflig@yahoo.com (F.F.); fpal@med.upatras.gr (F.P.); cgogos@med.upatras.gr (C.G.); 2 Department of Internal Medicine, University General Hospital of Patras, Rio, 265 04 Patras, Greece; 3 Division of Infectious Diseases, University General Hospital of Patras, Rio, 265 04 Patras, Greece; 4 Department for Infection Control, University General Hospital of Patras, Rio, 265 04 Patras, Greece; 5 School of Science and Technology, Hellenic Open University, 263 31 Patras, Greece; std528050@ac.eap.gr; 6 Department of Microbiology, University General Hospital of Patras, Rio, 265 04 Patras, Greece; i.siapika@gmail.com; 7 Department of Anesthesiology and Intensive Care Medicine, University General Hospital of Patras, Rio, 265 04 Patras, Greece; 8 3rd Department of Critical Care, Evgenidio Hospital, Medical School, National and Kapodistrian University of Athens, 115 28 Athens, Greece; gdimop@med.uoa.gr

**Keywords:** *Candida auris*, *Candidozyma auris*, bacteremia, candidemia, co-infection, MDR

## Abstract

Introduction: *Candida auris* (now *Candidozyma auris*) and multidrug-resistant (MDR) bacterial infections pose significant therapeutic challenges due to high antimicrobial resistance, increased mortality, and persistence in healthcare settings. In Greece, their rising prevalence is raising concerns regarding co-infection, yet comprehensive data remain limited. This study aims to investigate the epidemiology, risk factors, and clinical outcomes of MDR bacterial co-infection in patients with *C. auris* candidemia. Methods: This single-center, retrospective observational cohort study was conducted at a Greek tertiary university hospital and included adult patients with *C. auris* bloodstream infections from January 2019 to June 2024. The data were analyzed using appropriate statistical methodologies. Results: Among 96 patients, those with *C. auris* candidemia and MDR bacterial co-infection exhibited a significantly higher mortality rate (87.23% vs. 61.22%, *p* = 0.007). The presence of a central venous catheter was the only factor significantly associated with MDR co-infection (*p* = 0.030). In univariate analysis, MDR co-infection, a higher Charlson Comorbidity Index, and mechanical ventilation correlated with increased mortality. Multivariate analysis identified MDR co-infection (OR = 3.19, *p* = 0.045) and mechanical ventilation (OR = 7.07, *p* = 0.002) as independent mortality predictors. Conclusions: These findings underscore the need for enhanced surveillance, precise identification, and stringent infection control measures to prevent *C. auris* and MDR bacterial outbreaks in healthcare settings.

## 1. Introduction

*Candida auris* (now *Candidozyma auris*) is an emerging multidrug-resistant (MDR) fungal pathogen that has become a global health threat since its first description in 2009 [1]. It has been reported in over 61 countries across all six inhabited continents [2,3]. *C. auris* affects both adults and children with various risk factors, including immunosuppression, diabetes, recent antibiotic use, catheter use, and prolonged hospital stays [2,4,5]. The pathogen is difficult to identify using traditional methods and can be misidentified as other yeasts [4]. The fungus exhibits high resistance to fluconazole and moderate resistance to amphotericin B and caspofungin [2]. *C. auris* infections are associated with a 30-day mortality rate of 39.5%, with bloodstream infections having a higher mortality of 45% [2]. The pathogen’s ability to persist in hospital environments and resist decontamination procedures has led to outbreaks in healthcare facilities [6]. Hence, prompt investigation, aggressive interventions, and a robust response involving laboratories, clinicians, and public health agencies are crucial to manage cases and prevent transmission [7]

In this context, MDR bacterial infections pose a significant global health threat, with increasing prevalence and limited treatment options [8,9,10]. Antibiotic overuse in human therapies, animal husbandry, and aquaculture, as well as, poor antimicrobial stewardship and infection control practices [9], has contributed to rise of infections by MDR pathogens. The ESKAPE pathogens, including *Enterococcus faecium*, *Staphylococcus aureus*, *Klebsiella pneumoniae*, *Acinetobacter baumannii*, *Pseudomonas aeruginosa*, and *Enterobacter* spp., are particularly concerning, due to their role in life-threatening nosocomial infections [11]. Studies have identified various risk factors for MDR organisms, including diabetes mellitus, previous antibiotic use or hospitalization, use of medical devices, immunosuppressive therapy, and so on [12]. MDR infections are associated with increased morbidity, mortality, and healthcare costs [13]. Patients with MDR infections also had higher odds of 30-day readmission compared to those with non-MDR infections [14]. The rising prevalence of MDR infections presents challenges in selecting empiric antimicrobial therapy, necessitating a multidisciplinary approach involving antimicrobial stewardship and infection control practices to prevent further spread [9,10].

*C. auris* has emerged as a significant healthcare concern in Greece since its first isolation in 2019 [15]. The pathogen has rapidly spread across Greek healthcare facilities, with outbreaks reported in multiple hospitals [16,17]. *C. auris* isolates in Greece predominantly belong to the South Asian clade I and exhibit high resistance to fluconazole [16,18]. The fungus has become a leading cause of candidemia in some hospitals, with mortality rates exceeding 50% [16,17]. Of particular concern is the emergence of pan-echinocandin resistant isolates [17]. Similarly, Greece faces a significant challenge with MDR bacterial infections, particularly in hospital settings. A study of *P. aeruginosa* isolates from ventilator-associated pneumonia patients found high rates of MDR, extensively drug-resistant (XDR), and pandrug-resistant (PDR) strains, especially in Greece [19]. Recent surveillance data showed a rapid increase in MDR *P. aeruginosa* prevalence, rising by 63.8% from 2020 to 2023 [20]. MDR rates in Greece are among the highest in Europe, with carbapenem-resistant Gram-negative species becoming endemic in intensive care units [21]. A study of surgical ward patients found that 46.5% of isolated Gram-negative bacilli were MDR, with *A. baumannii* showing the highest MDR rate at 83.9% [22].

Of note, COVID-19 pandemic has led to profound shifts in healthcare delivery, with both immediate and lasting effects on individuals and communities. According to the CDC, contributing factors include a shift in patient demographics toward more severely ill individuals, reduced access to healthcare services leading to underdiagnosis and misdiagnosis, increased reliance on over-the-counter medications, and widespread antibiotic overuse. Additionally, challenges in laboratory supplies, diagnostic testing, and treatment availability have further complicated care. Crucially, the pandemic disrupted key infection prevention and control practices, such as hand hygiene, equipment disinfection, patient isolation, and consistent personal protective equipment use, ultimately undermining previous progress in addressing antimicrobial resistance (AMR). As a result, changes in hospitals’ daily practice due to the COVID-19 pandemic had a severe impact on AMR [23]. A reduction in the detection and reporting of AMR data was documented during the pandemic, along with an alarming increase in resistant infections during hospitalization [24,25,26,27]

Both *C. auris* and MDR bacterial pathogens have several common predisposing factors for colonization and invasive infections. However, there are limited data on co-infection, as well as, on associated patterns and outcomes. A recent report has noted a high rate of co-colonization with other MDR organisms among *C. auris* carriers, particularly in long-term acute care hospitals [28]; however, no information is available on co-infections. The primary objective of this study was to investigate the epidemiology of co-infection with MDR pathogens in patients with *C. auris* candidemia and to identify associated risk factors. The secondary objective included describing the clinical characteristics, antifungal susceptibility patterns, and outcomes of co-infection with MDR bacteria and *C. auris* candidemia.

## 2. Methods

### 2.1. Study Design, Patient Population, and Data Collection

This was a single-center, retrospective observational cohort study conducted in a Greek tertiary university hospital. Successive hospitalized adult patients diagnosed with bloodstream infection (primary or secondary) due to *C. auris* from 1 January 2019 to 1 June 2024, were included. Patient data were anonymously and securely recorded from medical records, including demographic characteristics, clinical parameters, laboratory values, microbiological data and outcomes. Patients with missing data files or evidence of commensal pathogens, indicative of contamination, were excluded from this analysis.

### 2.2. Microbiology Identification and Definitions

The initial identification of *C. auris* was performed through culture on chromogenic agar, followed by confirmation using MALDI-TOF MS (Bruker Daltonics, Billerica, MA, USA). Antifungal susceptibility testing was performed using the Etest gradient diffusion method (bioMérieux, Craponne, France). In *vitro* susceptibility of the bloodstream isolates was further evaluated using the Clinical and Laboratory Standards Institute (CLSI) reference broth microdilution method. The interpretation of resistance was based on the tentative breakpoints provided by the United States Centers for Disease Control and Prevention (CDC) for amphotericin B, fluconazole, and echinocandins, while species-specific CLSI epidemiological cut-off values were applied for the remaining azole antifungals [29,30].

Bacterial pathogens were identified using VITEK^®^ 2 identification cards (bioMérieux, Craponne, France). This study assessed susceptibility to various antimicrobial agents, as well as, the minimum inhibitory concentrations (MICs). Antimicrobials were chosen based on guidelines from the European Committee on Antimicrobial Susceptibility Testing (EUCAST) and recommendations from the European Centre for Disease Prevention and Control (ECDC) and the CDC. The MICs for colistin were determined using the broth microdilution method (SensiTest™ Colistin, Liofilchem, Italy), following EUCAST guidelines to ensure reliable results for this particular agent. Interpretation of the MIC values was conducted in accordance with the EUCAST standards applicable at the time of testing, categorizing isolates as susceptible, intermediate, or resistant. MDR was defined based on criteria from the ECDC and the CDC, as non-susceptible to at least one agent in three or more antimicrobial classes.

### 2.3. Management Protocols

Patients with suspected MDR bacterial infections were managed according to institutional protocols aligned with the Infectious Diseases Society of America (IDSA) and European Society of Clinical Microbiology and Infectious Diseases (ESCMID) guidelines [31,32]. Empiric antimicrobial therapy was initiated based on clinical presentation, the site of infection, history of prior antimicrobial therapy, tolerance or allergy, local resistance patterns, and infection severity. In cases of suspected or confirmed *C. auris*, initial antifungal therapy with echinocandins was employed [29]. Patients with confirmed or suspected MDR bacterial infections or *C. auris* were placed under strict contact precautions in single-patient rooms, including the use of gowns and gloves by all healthcare personnel upon room entry, with dedicated medical equipment where possible. Enhanced environmental cleaning protocols were implemented, especially for *C. auris*. All cases were evaluated by an infectious diseases specialist within 24 h of initiation of therapy. Upon availability of microbiological identification and antimicrobial susceptibility testing, targeted de-escalation was performed in accordance with antimicrobial stewardship principles to optimize treatment efficacy and minimize resistance development.

### 2.4. Statistical Methods

Categorical variables are presented as frequencies and relative frequencies, while continuous variables are presented as medians and interquartile ranges. Pearson’s chi-squared test (χ^2^) was used to assess associations between the categorical variables, with Yates’s correction for continuity applied where appropriate. In cases where the data structure did not permit the use of Pearson’s chi-squared test, Fisher’s exact test was employed.

Shapiro–Wilk and Kolmogorov–Smirnov tests were used to assess the normality of continuous variables. Normally distributed continuous variables were compared using Student’s t-test, whereas the Mann–Whitney U test was used for non-parametric comparisons.

In the logistic regression model for the multivariable evaluation of each independent variable’s effect, the area under the receiver operating characteristic (ROC) curve (AUC) was calculated to assess the model’s discriminative ability, with an AUC value greater than 0.7 considered acceptable. Additionally, pseudo R-squared statistics (McFadden’s R^2^, Cox & Snell R^2^, and Nagelkerke R^2^) were used to evaluate the model fit. Multicollinearity was assessed using the variance inflation factor (VIF), and a VIF < 5 was confirmed for all included variables. The selection of variables in the model was based on clinical relevance and statistical significance in univariable analysis.

All results are reported with 95% confidence intervals (95% CI). All tests were two-tailed, and statistical significance was set at *p* = 0.05.

Data analysis and visualization were performed in RStudio 2023.06.1, Build 524 (PBC, Boston, MA, USA), using the following R packages: “dplyr” v.1.1.4, “ggplot2” v.3.5.0, “scales” v.1.3.0, “nortest” v.1.0.4, “epiDisplay” v.3.5.0.2, “broom” v.1.0.5, “lmtest” v.0.9-40, “car” v.3.1-2, “stats” v.4.3.1, “ResourceSelection” v.0.3-6, “pROC” v.1.18.5, and “pscl” v.1.5.9.

### 2.5. Ethical Approval

The study was conducted according to the Declaration of Helsinki and Good Clinical Research Practice guidelines, and received approval from the local Ethics Committee and Institutional Review Board (391/31 July 2024). Informed consent was waived due to the retrospective nature of the study and the anonymous recording of data according to general data protection regulations.

## 3. Results

### 3.1. General Characteristics and Medical History of Participants

A total of 96 patients with candidemia (*C. auris*) were included in this study; their characteristics are shown in Table 1. Among the 96 hospitalized patients with candidemia, 48.96% (47 patients) also had a co-infection (at least one episode of bacteremia) with MDR bacterial pathogens. No statistically significant differences were observed between the two groups in terms of general characteristics, diagnosis on admission, or risk factors for MDR or *C. auris* infection/colonization. The only statistically significant difference was noted in the presence of a central venous catheter (CVC), which was more common among patients with co-infection by MDR pathogens (*p* = 0.030). The remaining risk factors did not show significant differences between the two groups.

As for *C. auris*, no difference in the resistance patterns or antifungal regimens used were noted between the groups (Supplementary Table S1). As expected, the majority of isolates were resistant to fluconazole (approximately 98%), followed by amphotericin B (24%), caspofungin (10%), anidulafungin (9%), and voriconazole (8%). More than 87% of cases were treated with anidulafungin or micafungin, while approximately 4% required therapy with amphotericin B, either alone or in combination with echinocandin.

Among the participants with co-infection by MDR pathogens, 89.36% of the infections were monomicrobial, whereas 10.64% were polymicrobial (Supplementary Table S2). From the total number of isolated pathogens (n = 52), 34.04% were Enterococci, of which 93.75% were vancomycin-resistant (VRE). Other isolated pathogens included *Acinetobacter* spp. (23.4%), *Klebsiella pneumoniae* (19.15%), *Pseudomonas* spp. (12.77%), *E. coli* (4.26%), other *Candida* spp. (12.77%), and miscellaneous pathogens (4.26%). Regarding antimicrobial resistance, carbapenemase-producing *K. pneumoniae* (KPC) was detected in 7.69% of cases. Additionally, metallo-β-lactamases (NDM, VIM) were identified in approximately 15% of the isolated strains. In contrast, extended-spectrum β-lactamases (ESBLs) were not detected (Supplementary Table S2).

### 3.2. Laboratory Findings

The laboratory parameters did not exhibit any statistically significant differences between the participant groups (Supplementary Table S3).

### 3.3. Outcomes

Regarding patient outcomes, the participants with concomitant co-infection with MDR pathogens exhibited a statistically significantly higher mortality rate (87.23% vs. 61.22%, *p* = 0.007). Conversely, post-infection colonization was marginally more frequent in the group with isolated candidemia (22.45% vs. 6.38%, *p* = 0.05). No other significant differences in outcome characteristics were observed between the two groups (Table 2).

### 3.4. Candidemia with MDR Co-Infection as an Independent Mortality Risk Factor

The potential associations between mortality outcomes and candidemia with MDR co-infection, age, length of hospital stay (LOS), CCI, and invasive mechanical ventilation (IMV) were analyzed. In the univariate analysis, candidemia with MDR co-infection was significantly associated with an increased risk of mortality (OR = 4.33, 95% CI: 1.54–12.14, *p* = 0.005). Additionally, a higher CCI score (OR = 1.29, 95% CI: 1.01–1.65, *p* = 0.038) and IMV (OR = 4.73, 95% CI: 1.74–12.86, *p* = 0.002) were also significantly correlated with mortality. Age exhibited a borderline but non-significant association (OR = 1.03, 95% CI: 1–1.06, *p* = 0.062), while LOS was not statistically significant (*p* = 0.529). In the multivariate analysis, candidemia with MDR co-infection remained an independent predictor of mortality (OR = 3.19, 95% CI: 1.03–9.9, *p* = 0.045), along with IMV, which showed a strong independent association (OR = 7.07, 95% CI: 2.03–24.67, *p* = 0.002). Conversely, age (*p* = 0.864), LOS (*p* = 0.809), and CCI (*p* = 0.137) did not retain statistical significance after adjustment. These findings, summarized in Table 3, provide insight into both univariate and multivariate analyses, highlighting the significant role of MDR co-infection and intubation in patient mortality.

## 4. Discussion

This study aimed to investigate the patterns of MDR pathogen bacteremia in patients with *C. auris* candidemia, identify associated risk factors, and assess their impact on clinical outcomes. Patients with *C. auris* candidemia, with and without MDR co-infection, exhibited comparable characteristics, except for the presence of CVCs, which was more prevalent in those with MDR co-infections. Mortality rates were significantly higher in patients with MDR co-infections, even though *C. auris* colonization occurred less frequently after the initial infection. IMV and co-infection with MDR pathogens were identified as significant predictors of increased mortality in patients with *C. auris* candidemia.

Our study is one of the largest cohorts of *C. auris* patients described in the global literature. Studies have reported outbreaks in Oman [33], the UK [34], and the USA [35], highlighting its global spread. The COVID-19 pandemic has led to an increase in *C. auris* infections, particularly among critically ill patients in ICUs [36,37]. *C. auris* has emerged as a significant threat in healthcare settings, with studies reporting it as the most isolated *Candida* species in blood cultures of COVID-19 patients [37]. Prior data indicate an increasing trend in *C. auris* colonization and clinical infections, particularly within long-term care settings, such as long-term acute care hospitals and ventilator-equipped skilled nursing facilities [38,39]. Key risk factors for *C. auris* colonization and candidemia include IMV, CVC, total parenteral nutrition, and prolonged ICU stays [40,41,42]. Prior antibiotic use, especially carbapenems, and antifungal exposure, particularly fluconazole, are also associated with increased risk [39,42]. Almost all our patients had priorly received antibiotics, whereas the majority had been recently hospitalized or were under invasive mechanical ventilation in our cohort.

Nearly half of the patients with *C. auris* candidemia also had MDR bacteremia. Concurrent fungemia and bacteremia represents a serious condition associated with critically ill patients, particularly in surgical settings. Studies have shown that polymicrobial fungemia accounts for 3.4% of fungemia cases, with *Candida* species being the most common fungal pathogens [43,44]. Predisposing factors include intravascular catheters, antibiotic use, and surgical procedures [44], while mixed septicemia, characterized by synchronous bacterial and fungal bloodstream infections, is a marker for critically ill surgical patients with high mortality rates [45]. The 50% incidence of MDR pathogens observed in this study is consistent with the high prevalence of MDR infections in Greece, as reported in previous studies, [21,46] and a recent report by the European Centre for Disease Prevention and Control (ECDC) [47]. Since the late 2000s, Greece has faced an endemic challenge of MDR pathogens within its hospital sector, predominantly driven by carbapenem-resistant Gram-negative bacilli; the country consistently reports some of the highest antimicrobial resistance rates in Europe [47].

Patients with *C. auris* infection, with or without MDR co-infection, did not differ in terms of risk factors. This is in line with previous data showing common risk factors for *C. auris* infection or colonization and MDR bacteremia, including the prior use of antibiotics, immunocompromise, indwelling catheters, IMV, and prior recent hospitalization [3,12,39]. However, patients with MDR co-infections had a higher prevalence of CVC use (87.23 vs. 66.67%). The presence of a CVC is associated with higher rates of bloodstream infections [48,49]. Central line-associated bloodstream infections (CLABSIs) are a significant healthcare concern, with MDR organisms causing up to 67% of cases [50]. Gram-negative bacteria are the predominant pathogens in some settings, including Greece, Taiwan, Malaysia, Saudi Arabia, and China, with 9% being MDR pathogens [51,52,53,54,55]. CLABSIs are associated with increased mortality, costs, and readmission rates. Failure to promptly remove CVC in cases of CLABSI caused by MDR pathogens has been strongly associated with increased 30-day mortality, as persistent infection and inadequate source control can significantly worsen patient outcomes [56]. Therefore, the implementation of standardized central line care bundles, including the proper insertion techniques, regular line maintenance, and timely removal when no longer necessary, remains a critical strategy to minimize the risk of CLABSI, particularly in high-risk populations vulnerable to MDR infections [51].

In our report, *C. auris* colonization was observed in approximately 40% and 15% of patients, prior to and following candidemia, respectively. Research indicates that *C. auris* colonization is prevalent in healthcare settings, especially among critically ill patients and residents of long-term care facilities [39,57,58]. *C. auris* can colonize multiple body sites, with the axilla and groin being common locations [57,59]. The persistence of *C. auris* on skin and environmental surfaces contributes to its transmission in healthcare settings [59]. Outbreaks have been associated with contaminated medical equipment, such as reusable temperature probes [60]. Effective control measures involve comprehensive patient and environmental screening, chlorhexidine washes for patients, and thorough surface disinfection with suitable agents, such as stabilized hydrogen peroxide [61]. When followed correctly, proper hand hygiene and commonly used disinfectants can be effective against *C. auris* [61]. Sodium hypochlorite isotonic solution has shown potential for patient decolonization [62]. However, *C. auris* can survive on dry fabrics for up to seven days, posing challenges for eradication [61]. Colonization with *C. auris* significantly increases the risk of subsequent invasive infection, often by the same strain [63]. We observed reduced post-infection colonization in patients with MDR co-infection. It is possible that, enhanced disinfectant and de-colonization measures associated with MDR bacteremia may have contributed to the (marginally) significant difference between the groups. However, this finding should be interpreted with caution. The CDC no longer recommends routine reassessments for *C. auris* colonization, partly due to the fluctuating nature of its detection, as *C. auris* can sometimes be present and at other times undetected, especially in healthcare settings [38]. To manage *C. auris* in healthcare settings, rigorous colonization screening and strict infection control measures are essential [64].

*C. auris* exhibits high resistance to fluconazole and moderate resistance to amphotericin B and caspofungin, while remaining sensitive to echinocandins, like micafungin and anidulafungin [2,65]. This resistance is attributed to multiple molecular mechanisms. Mutations in the ERG11 gene, increased copy numbers of this gene, and the overexpression of efflux pumps contribute to fluconazole resistance [66]. A novel genetic determinant, mutations in the TAC1B gene, has been identified as a significant contributor to clinical fluconazole resistance [67]. These mutations can arise rapidly upon exposure to fluconazole and are present in the majority of fluconazole-resistant *C. auris* isolates globally. Additionally, resistance to other antifungals has been observed, including amphotericin B (10–30% of strains) and echinocandins (up to 5% of strains) [68]. We also recorded high resistance to fluconazole in our study, in line with the fact that the majority of patients had been under antifungal treatment in the past.

The majority of patients are empirically treated with echinocandins, in accordance with international guidelines and local protocols [29]. This regimen is later modified if required, following antifungal sensitivity testing and patient individual needs, e.g., tolerance, allergies, drug–drug interactions etc. At the moment, *C. auris* commonly shows resistance to fluconazole and amphotericin B, with echinocandin resistance emerging in some regions [69]. In this context, several new antifungals are being developed to combat *C. auris*. APX001A, which inhibits the fungal protein Gwt1, demonstrated potent in vitro activity and improved survival in mouse models compared to anidulafungin [70]. Ibrexafungerp, a novel oral triterpenoid antifungal, showed broad *in vitro* activity against *C. auris*, including isolates with fks mutations, and promising clinical results in two patients [71]. Other investigational agents include rezafungin, manogepix/fosmanogepix, olorofim, and tetrazoles, which have shown improvements in survival and reduction in tissue fungal burden in animal models [72].

The overall mortality rate for *C. auris* infections ranges from 30% to 60%, with bloodstream infections having a 45% mortality rate [2]. However, a recent study suggests that mortality may be lower when adjusted for confounding variables [73]. In our cohort, mortality was approximately 60% in patients with *C. auris* candidemia. We found significantly increased mortality in *C. auris* patients with MDR co-infections compared to those without (87%), while MDR infection was identified as an independent risk factor for mortality. This finding is consistent with previous research, including ours, demonstrating increased mortality rates among patients with MDR infections [74,75,76]. However, it has been suggested that the elevated mortality risk associated with MDR pathogens is primarily attributed to the delayed initiation of appropriate antimicrobial therapy, rather than the intrinsic virulence of the resistant microorganisms themselves [74,77,78,79]. Even though the treatment protocols at our site align well with international guidelines on the management of MDR pathogens (see methods), no information is available on the details of the regimens and the timing used.

Invasive ventilation was identified as an independent predictor for mortality in these patients. Invasive mechanical ventilation in critically ill patients is strongly associated with high mortality rates, particularly in pediatric hematopoietic stem cell transplant recipients, with reported pediatric ICU mortality rates as high as 60.4% [80]. In patients with COVID-19-related acute respiratory distress syndrome (ARDS), factors such as elevated driving pressure and ventilatory rate have been identified as key contributors to increased mortality [81]. For patients without ARDS, maximum airway pressure has emerged as the primary respiratory factor, independently linked to in-hospital mortality [82]. Furthermore, early extubating to non-invasive ventilation as part of a weaning strategy has been shown to reduce hospital mortality, particularly in patients with chronic obstructive pulmonary disease. This strategy contributes to a reduction in the duration of invasive ventilation and ICU length of stay, associated with a lower incidence of ventilator-associated pneumonia [83].

Despite being one of the largest *C. auris* cohorts described in the literature, our study suffers inherent limitations. First it was a retrospective single-center study; hence, our findings and conclusions drawn should be interpreted with caution. Diverse practices and increased local MDR endemicity limits generalization of our results. Moreover, misidentification of *C. auris* is common due to inadequate reference databases on diagnostic platforms [84]. Additionally, the correlation between MIC values and clinical outcomes is poorly understood, resulting in a lack of *C. auris*-specific breakpoints [69]. Although various clinical outcomes, such as mortality, LOS, and ICU admission, were assessed, this study lacked crucial information on several key variables that could significantly influence these endpoints. Specifically, no detailed data were available regarding the antibacterial treatment regimens employed, including type, dosage, duration, and timing of administration. Additionally, the absence of information on source control measures, such as surgical interventions, drainage procedures, and institutional disinfection practices, limits our ability to interpret the results fully. These factors are known to play a critical role in patient outcomes and may confound the relationship between the observed variables and the reported clinical outcomes.

Our study was the first to assess *C. auris* and bacterial MDR co-infection. No specific factors were identified in the *C. auris* patient group also presenting with MDR pathogens, except for the presence of CVC. MDR co-infections were associated with increased mortality, especially among patients under IMV. Our findings underscore the critical need for enhanced infection control measures in healthcare settings. Close monitoring and stringent catheter care protocols are essential, particularly among high-risk patients, while targeted antimicrobial stewardship, early diagnostic screening, and rigorous hygiene practices should be prioritized. Healthcare personnel must be regularly trained in recognizing and managing co-infections, while infection prevention teams should consider implementing routine surveillance for MDR pathogens in patients with *C. auris*. This can be challenging in low- or middle-income countries, while global mobility adds to the complexity, facilitating the cross-border spread of *C. auris* and MDR organisms. Global health bodies and national public health authorities must invest in building laboratory capacity, enforcing standardized infection prevention and control guidelines, and supporting antimicrobial stewardship programs across all healthcare levels. International collaboration, real-time data sharing, and coordinated surveillance efforts are essential to monitor trends, detect outbreaks early, and mount effective, evidence-based responses to this dual threat.

## 5. Conclusions

Our findings underscore the urgent need for enhanced surveillance, accurate identification methods, and stringent infection control measures to combat *C. auris,* prevent new infections with MDR pathogens and avoid epidemic outbreaks.

## Figures and Tables

**Table 1:** General characteristics and medical history of participants.

	Candidemia		
	Without Co-Infection (n = 49)	With MDR Co-Infection (n = 47)	*p*
GENERAL CHARACTERISTICS			
Age (Years)	69 (57–76)	70 (64–77)	0.392
Gender (Male)	26 (53.06 %)	30 (63.83 %)	0.388
CCI	4 (3–5)	4 (3–6)	0.372
Sepsis	12 (24.49%)	12 (25.53 %)	1
DIAGNOSIS			
COVID-19	8 (16.33%)	10 (21.28%)	0.719
RTI	18(36.73%)	17(36.17%)	1
UTI	4(8.16%)	3(6.38%)	1
Cerebrovascular Events	9(18.37%)	8(17.02%)	1
Cardiovascular Events	1(2.04%)	1(2.13%)	1
Trauma	8(16.33%)	6(12.77%)	0.837
Other	1(2.04%)	2(4.26%)	0.481
RISK FACTORS for MDR/*C. auris* Infection			
CVC	32 (66.67%)	41 (87.23%)	0.030
Foley Catheter	43 (87.76%)	44 (93.62%)	0.487
PICC	22 (44.9 %)	17 (36.17%)	0.507
IMV	24 (48.98%)	29 (61.7%)	0.294
Non-IMV	1 (2.04%)	2 (4.26%)	0.613
History of Prior Antifungal Treatment	28 (57.14%)	21 (44.68%)	0.292
History of Prior Antibiotic Treatment	47 (95.92%)	45 (95.74%)	1
Recent Hospitalization, within 6 Months (Yes)	34 (69.39%)	30 (63.83 %)	0.718
Pre-Existing *Candida* Colonization	21 (42.86%)	19 (40.43%)	0.972

CCI: Charlson Comorbidity Index; RTI: respiratory tract infections; UTI: urinary tract infections; MDR: multidrug-resistant pathogen; CVC: central venous catheter; PICC: peripherally inserted central catheter; IMV: invasive mechanical ventilation.

**Table 2:** Outcomes of participants.

		Candidemia		
		Without Co-Infection (n = 49)	With MDR Co-Infection (n = 47)	*p*
Intensive Care Unit Admission (Yes)		23 (46.94%)	25 (53.19%)	0.683
Mortality		30 (61.22%)	41 (87.23%)	0.007
	28-Day Mortality	19 (63.33%)	28 (68.29%)	0.066
	90-Day Mortality	11 (36.67%)	13 (31.71%)	0.723
	Death Associated with *C. auris*	27 (90%)	34 (82.93%)	0.501
Candidemia Resolution		38 (77.55%)	30 (63.83%)	0.209
Time to Bloodstream *Candida* Clearance (Days)		5 (2–9.75)	6 (3.25–10)	0.464
*C. auris* Relapse		16 (32.65%)	11 (23.4%)	0.435
Post-Infection *Candida* Colonization		11 (22.45%)	3 (6.38%)	0.050
LOS (Days)		68 (30–130)	46 (32.5–105.5)	0.465

MDR: Multidrug-resistant pathogens; LOS: length of stay.

**Table 3:** Univariate and multivariate analysis of factors associated with mortality risk.

	Unadjusted Analysis			Adjusted Analysis		
	Odds Ratio	95% CI	*p*-Value	Odds Ratio	95% CI	*p*-Value
Candidemia Status (with MDRs)	4.33	1.54–12.14	**0.005**	3.19	1.03–9.9	0.045
Age	1.03	1–1.06	0.062	1.005	0.95–1.07	0.864
LOS	1.00	1–1.01	0.529	1	0.99–1.01	0.809
CCI	1.29	1.01–1.65	**0.038**	1.43	0.89–2.29	0.137
IMV (Yes)	4.73	1.74–12.86	**0.002**	7.07	2.03–24.67	0.002

MDR: Multidrug-resistant pathogens; LOS: length of stay; CCI: Charlson Comorbidity Index; IMV: invasive mechanical ventilation.

## Data Availability

The original contributions presented in this study are included in the article/Supplementary Materials. Further inquiries can be directed to the corresponding author.

## Supplementary Materials

The following supporting information can be downloaded at: https://www.mdpi.com/article/10.3390/jof11060407/s1, Table S1. *C. auris* resistance patterns and employed therapy. Table S2. Microbiological profile and antimicrobial resistance in patients with candidemia and MDR co-infection. Table S3. Laboratory parameters.

## Abbreviations

MDR	Multidrug-resistant
PDR	Pandrug-resistant
AMR	Antimicrobial resistance
CVC	Central venous catheter
RTI	Respiratory tract infections
UTI	Urinary tract infections
VRE	Vancomycin-resistant Enterococci
ESBLs	Extended-spectrum β lactamases
KPC	*K. pneumoniae* carbapenemase
MBL	Metallo-β-lactamases
NDM	New Delhi metallo-β-lactamase
VIM	Verona integron-encoded metallo-β-lactamase
LOS	Length of stay
CCI	Charlson Comorbidity Index
IMV	Invasive mechanical ventilation
ECDC	European Centre for Disease Prevention and Control
CLABSIs	Central line-associated bloodstream infections
ICU	Intensive care unit
ARDS	Acute respiratory distress syndrome
