# Supplementary material for: Patterns and Predictors of Candida auris Candidemia with Multidrug-Resistant Bacterial Co-Infections: Results from the CANDI-MDR Study
Source: J Fungi (Basel). 2025 May 25;11(6):407. doi: 10.3390/jof11060407 (PMC12194493; doi:10.3390/jof11060407)
Supplement: Supplementary file 1 [file jof-11-00407-s001.zip › jof-3600270-supplementary.pdf]

Table S1. *C. auris* resistance patterns and employed therapy.

	Candidemia		
	**Without Co-Infection (n = 49)**	**With MDR Co-Infection (n = 47)**	***p***
***C. auris* ANTIFUNGAL RESISTANCE**			
**Fluconazole**	48 (97.96%)	47 (100%)	1
**Itraconazole**	1 (2.04 %)	0 (0.0%)	1
**Amphotericin**	12 (24.49%)	11 (23.4%)	1
**Voriconazole**	5 (10.2%)	3 (6.38%)	0.715
**Anidulafungin**	6 (12.24%)	3 (6.38%)	0.487
**Caspofungin**	7 (14.29%)	3 (6.38%)	0.317
**Micafungin**	2 (4.08%)	0 (0.0%)	0.495
***C. auris* ANTIFUNGAL THERAPY**			
**Anidulafungin**	28(57.14%)	27(57.45%)	1
**Micafungin**	15(30.61%)	15(31.92%)	0.723
**Amphotericin (alone or in combination with echinocandin)**	3(6.12%)	1(2.13%)	0.489
**Fluconazole**	2(4.08%)	1(2.13%)	1
**Treatment Not Documented**	1(2.04%)	3(6.38%)	0.356

MDR: Multidrug-resistant pathogen.

Table S2. Microbiological profile and antimicrobial resistance in patients with candidemia and MDR co-infection.

	**Candidemia with MDR Co-Infection (n = 47)**
**IDENTIFIED MDR BACTERIAL PATHOGENS**	
**Number of Pathogens (excluding *C. auris*)**	
Monomicrobial Infection	42 (89,36%)
Polymicrobial Infection	5 (10,64%)
**Types of Isolated Pathogens**	**n = 52**
E. coli	2 (4,26%)
Pseudomonas spp.	6 (12.77 %)
Klebsiella pneumoniae	9 (19.15 %)
Acinetobacter spp.	11 (23.4 %)
Enterococci	16 (34.04 %)
VRE	15 (93.75%)
Candida spp. (excluding *C. auris*)	6 (12.77 %)
Other Pathogens	2 (4,26%)
**IDENTIFIED MECHANISMS OF ANTIMICROBIAL RESISTANCE (% of isolated pathogens)**	
**KPC**	4 (7.69 %)
**MBL**	
**NDM**	6 (11.5%)
**VIM**	2 (3.85%)
**ESBL**	0 (0%)

MDR: Multidrug-resistant pathogen; VRE: vancomycin-resistant Enterococci; KPC: *K. pneumoniae* carbapenemase; MBL: metallo-β-lactamases; NDM: New Delhi metallo-β-lactamase; VIM: Verona integron-encoded metallo-β-lactamase; ESBL: extended-spectrum β-lactamase.

Table S3. Laboratory parameters.

	Candidemia		
	**Without Co-Infection (n = 49)**	**With MDR Co-Infection (n = 47)**	***p***
**LABORATOTY PARAMETERS**			
**WBC (×10⁹/L)**	9.07 (5.84–11.96)	9.68 (6.92–13.43)	0.716
**PMN (%)**	75.6 (60.3–82.4)	76.2 (57.55–88.3)	0.597
**Lymphocytes (%)**	13.2 (8.5–21.5)	13.2 (6.6–23.65)	0.889
**Hemoglobin (g/dL)**	8.6 (7.8–9.5)	8.8 (8.05–8.944)	0.610
**Platelets (PLT ×10⁹/L)**	152 (63–252)	153 (51–236)	0.770
**Total Bilirubin (mg/dL)**	0.550 (0.4–0.82)	0.70 (0.5–1.185)	0.060
**Creatinine (mg/dL)**	0.9 (0.5–1.40)	0.8 (0.55–1.50)	0.453
**LDH (U/L)**	252 (196–338)	292 (205–392)	0.310
**CRP (mg/L)**	8.14 (3.17–15.7)	7.79 (3.06–14.21)	0.848
**ESR (mm/h)**	52 (26.5–116)	79.5 (65.5–114.5)	0.297
**Ferritin (ng/mL)**	1283 (408.5–2667)	1243 (420.5–2486.5)	0.778

MDR: Multidrug-resistant pathogen; WBC: white blood cell; PMN: polymorphonuclear leukocyte; PLT: platelets; CRP: C-reactive protein; ESR: erythrocyte sedimentation rate.
